# Identification of SUMO Proteins and Their Expression Profile During Induction of Somatic Embryogenesis in *Medicago truncatula* Gaertn.

**DOI:** 10.3390/ijms26178133

**Published:** 2025-08-22

**Authors:** Anna Kujawska, Paulina Król

**Affiliations:** Institute of Biology, University of Szczecin, Wąska 13, 71-415 Szczecin, Poland

**Keywords:** SUMO, SUMOylation, epigenetic, somatic embryogenesis, legume, *Medicago truncatula*

## Abstract

Somatic embryogenesis (SE) is a key plant regeneration technique involving the reprogramming of somatic cells into embryogenic structures. This developmental transition is regulated by complex genetic and epigenetic mechanisms, including post-translational modifications such as SUMOylation—the covalent attachment of small ubiquitin-like modifier (SUMO) proteins to target proteins, influencing their function, stability, and interactions. While SUMOylation is known to regulate plant development and stress responses, its role in SE has remained unknown. In this study, we investigated the involvement of the SUMOylation pathway in SE induction in *Medicago truncatula*. Using BLASTp analysis with known SUMO pathway proteins from *Arabidopsis thaliana* and *Glycine max*, we identified 10 homologous genes in *M. truncatula*. Phylogenetic relationships, gene structure, and conserved motif analyses confirmed their evolutionary conservation and characteristic domains. Expression profiling revealed significant upregulation of SUMO pathway genes—including Mt *SUMO2*, Mt *SAE1-2*, Mt *SCE1a-b*, Mt *MMS21*, and Mt *PIAL2*—in embryogenic cell lines during early SE induction. Additionally, in silico prediction of SUMOylation sites and SUMO-interacting motifs (SIMs) in 12 key SE regulatory proteins indicated a broad potential for SUMO-mediated regulation. These findings suggest that SUMOylation may contribute to the acquisition of embryogenic competence during somatic cell reprogramming in plants.

## 1. Introduction

Somatic embryogenesis (SE) is a complex developmental process in which somatic cells of plant explants undergo a series of morphogenetic and physiological changes, ultimately leading to the formation of bipolar structures known as somatic embryos. These embryos have the potential to develop into fully functional plants. Unlike zygotic embryogenesis, which depends on fertilization, SE is an asexual process in which embryos develop without gamete fusion [1,2]. SE can occur either directly from explant cells (direct SE, DSE) or indirectly via an intermediate callus phase (indirect SE, ISE). This process relies on cellular totipotency, intricate signaling networks, and gene expression changes at both genetic and epigenetic levels, which regulate transitions through distinct developmental stages—such as induction of pro-embryogenic masses (PEMs), embryo formation, maturation, and plant regeneration.

Epigenetic regulation, defined as heritable changes in gene expression that do not alter the DNA sequence, plays a key role in controlling plant developmental processes, including SE. Dynamic modifications such as DNA methylation, histone alterations, and non-coding RNA-mediated gene silencing contribute to transcriptional reprogramming. During SE, these epigenetic modifications—often triggered by stress or the presence of exogenous plant growth regulators (PGRs) in the culture medium—can activate genes involved in embryogenesis while repressing those related to differentiation and maintenance of somatic identity (reviewed in [3,4]).

Studies in *Medicago truncatula*, a model legume species, have shown that epigenetic reprogramming is crucial for the induction of embryogenic callus and subsequent somatic embryo development. Key components of this process include DNA methylation [5] and post-translational histone modifications mediated by regulators such as PICKLE (PKL) [6], Polycomb group (PcG), and Trithorax group (TrxG) proteins [7,8].

One emerging mechanism potentially involved in this regulation is SUMOylation—a reversible post-translational modification mediated by small ubiquitin-like modifier (SUMO) proteins. SUMOylation modulates protein activity, stability, subcellular localization, and interactions with other proteins or DNA. In plants, this modification has been implicated in diverse environmental stress responses—including phosphate deficiency, heat, cold, drought, and pathogen defense [9,10,11,12,13,14,15]—as well as in essential physiological processes such as cell growth and development, seedling establishment, root architecture, nitrogen assimilation, and flowering time (reviewed in [16]).

SUMOylation is a three-step, ATP-dependent enzymatic process. In the first step, SUMO precursor proteins are processed by specific proteases to expose a conserved C-terminal diglycine (GG) motif, generating mature SUMO. This is followed by a cascade of reactions involving a SUMO-activating enzyme (E1; SAE1/SAE2), a SUMO-conjugating enzyme (E2; SCE1), and a SUMO ligase (E3). In *Arabidopsis thaliana*, four E3 ligases have been identified: SAP AND MIZ1 DOMAIN-CONTAINING LIGASE 1 (SIZ1) [9], METHYL METHANESULFONATE-SENSITIVITY PROTEIN 21 (MMS21) [17], and PROTEIN INHIBITOR OF ACTIVATED STAT-LIKE 1 and 2 (PIAL1–2) [18].

The E1 enzyme activates SUMO by adenylating its C-terminal glycine in an ATP-dependent reaction, which then forms a thioester bond by transferring SUMO to a catalytic cysteine residue within the E1 enzyme. In the second step, SUMO is transferred to a cysteine residue in the catalytic site of the E2 enzyme, forming a new SUMO–E2 thioester bond. Subsequently, in the presence of an E3 ligase, the E2 enzyme facilitates the covalent attachment of SUMO to the ε-amino group of lysine residues on the target protein. E3 ligases enhance the efficiency and specificity of SUMOylation by simultaneously interacting with the E2 enzyme, the SUMO protein, and the substrate, and by properly orienting the E2–SUMO complex for optimal transfer. Protein substrates can be SUMOylated at single or multiple lysine residues and may also undergo poly-SUMOylation, mediated by E4 ligases [18]. SUMO-specific proteases reverse this modification by cleaving the isopeptide bond between SUMO and its target, maintaining the dynamic balance between free and conjugated SUMO forms.

Although SUMOylation typically targets lysine residues within the ψKxD/E consensus sequence (Ψ = hydrophobic residue), 20–25% of events occur outside this motif, though ~80% of SUMOylated proteins in *Arabidopsis* contain it [19,20]. A major function of SUMOylation is to promote protein–protein interactions, often via a SUMO-interacting motif (SIM) on the binding partner [21]. The SIM, typically composed of three hydrophobic amino acids and an adjacent acidic region, facilitates binding to SUMO proteins and is widely present across various proteins.

In *Arabidopsis*, SUMOylation is essential for zygotic embryogenesis; mutations in key components of the pathway, such as *SAE2* and *SCE1*, result in embryo lethality at early developmental stages. Additionally, SUMO isoforms exhibit tissue- and stage-specific expression patterns: At *SUMO1* and At *SUMO2* are active during embryogenesis but display non-overlapping localization, while At *SUMO3* is largely absent from embryonic tissues and is instead expressed predominantly in floral organs [22,23].

Despite extensive research on SUMOylation in various aspects of plant development, its role in somatic embryogenesis (SE) remains largely unexplored. *Medicago truncatula* is a valuable model for studying developmental and molecular processes due to its well-established SE protocols and rich genetic resources. In this study, we aimed to (i) identify genes encoding SUMO pathway components in the *M. truncatula* genome and (ii) examine their expression during the induction phase of SE in embryogenic (M9-10a) and non-embryogenic (M9) lines. Our findings provide new insights into the potential involvement of SUMOylation in the transition from somatic to embryogenic cell fate in *M. truncatula*, laying the groundwork for further investigations into epigenetic regulation of this process.

## 2. Results

### 2.1. Characterization and Identification of SUMO Pathway Proteins in Medicago truncatula

To investigate the potential role of SUMOylation in somatic embryogenesis (SE) in *Medicago truncatula*, we first performed a comprehensive identification and characterization of genes encoding components of the SUMOylation pathway. Using BLASTp searches with known SUMO-related protein sequences from *Arabidopsis thaliana* and *Glycine max*, we identified 10 SUMOylation-related genes in the *M. truncatula* genome. These genes were classified into four major groups based on their predicted functions: SUMO proteins, E1 ligases, E2 conjugating enzymes, and E3 ligases (Table 1).

**Figure 1:**
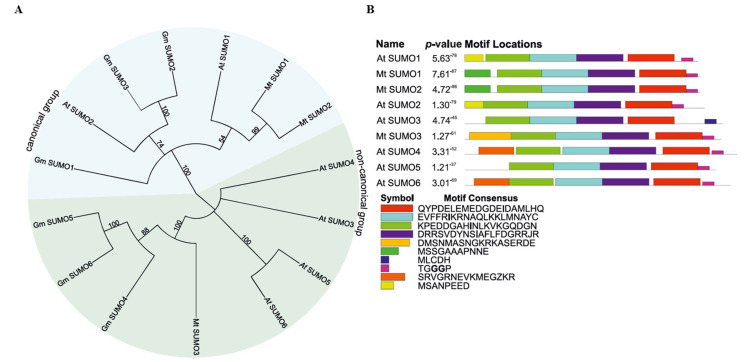
Phylogenetic tree (**A**) and conserved motifs of identified SUMO proteins in *Medicago truncatula* (**B**). (**A**) Protein sequences from *Arabidopsis thaliana* (*At*), *Glycine max* (*Gm*), and *M. truncatula* (*Mt*) were used to construct a phylogenetic tree using the Neighbor-Joining method. Numbers at each node represent bootstrap values. (**B**) The *p*-values for the protein motifs represent the statistical significance of the motif occurrence.

To elucidate the evolutionary relationships among SUMO proteins from *M. truncatula*, *Arabidopsis thaliana*, and *Glycine max*, a Neighbor-Joining phylogenetic tree was constructed (Figure 1A). The SUMO family in *M. truncatula* comprises three members, designated Mt SUMO1, Mt SUMO2, and Mt SUMO3. Although the genome contains genes encoding these three proteins, we were unable to identify additional homologs found in other species. The SUMO proteins segregated into two distinct clades: a canonical group, including At SUMO1-2, Gm SUMO1-3, and Mt SUMO1-2; and a non-canonical group, comprising At SUMO3-6, Gm SUMO4-6, and Mt SUMO3. Notably, Mt SUMO1 and Mt SUMO2 exhibit closer phylogenetic affinity to At SUMO1 than to At SUMO2 or Gm SUMO1-3 proteins. Conversely, Mt SUMO3 clusters with Gm SUMO4-6 within the non-canonical clade. All Mt SUMO proteins possess the conserved diglycine motif at their C-terminus (Figure 1B).

**Figure 2:**
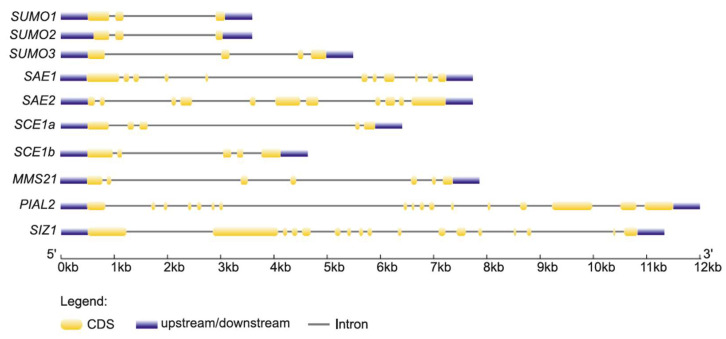
Gene structure of the identified SUMO pathway members in *Medicago truncatula* Gaertn.

The gene structures of SUMO in *M. truncatula* were examined, focusing on conserved motifs as well as the distribution of introns and exons within the sequences. The analysis revealed clear differences between the two groups of Mt *SUMO*: canonical *SUMO* genes contain three exons, whereas non-canonical genes consist of four exons (Figure 2). MEME analysis detected conserved motifs shared by all SUMO proteins from *M. truncatula* and *A. thaliana*, further supporting the phylogenetic relationships presented in Figure 1B.

**Figure 3:**
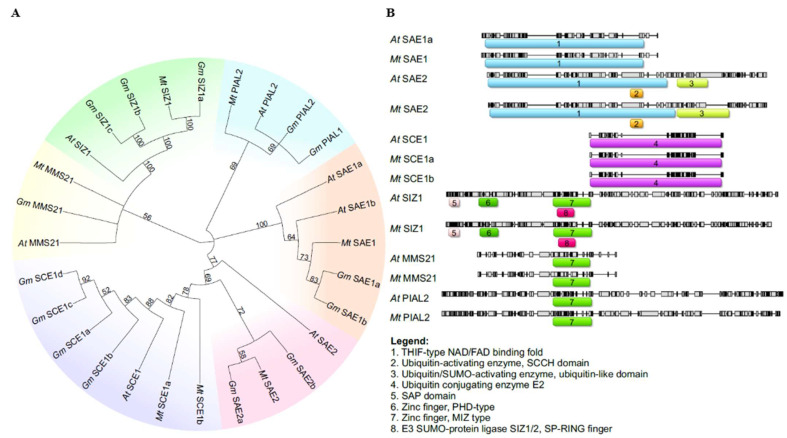
Phylogenetic tree (**A**) and a domain location schematic view of SUMO enzyme proteins in *Medicago truncatula* (**B**). Protein sequences from *Arabidopsis thaliana* (*At*), *Glycine max* (*Gm*), and *M. truncatula* (*Mt*) were used to construct a phylogenetic tree using the Neighbor-Joining method. Colors in the phylogenetic tree indicate protein families: SAE1/SAE2 (orange/pink), SCE1 (purple), SIZ1 (green), MMS21 (yellow), and PIAL2 (blue). Colors in the domain schematic are used only to visually distinguish domains.

**Figure 4:**
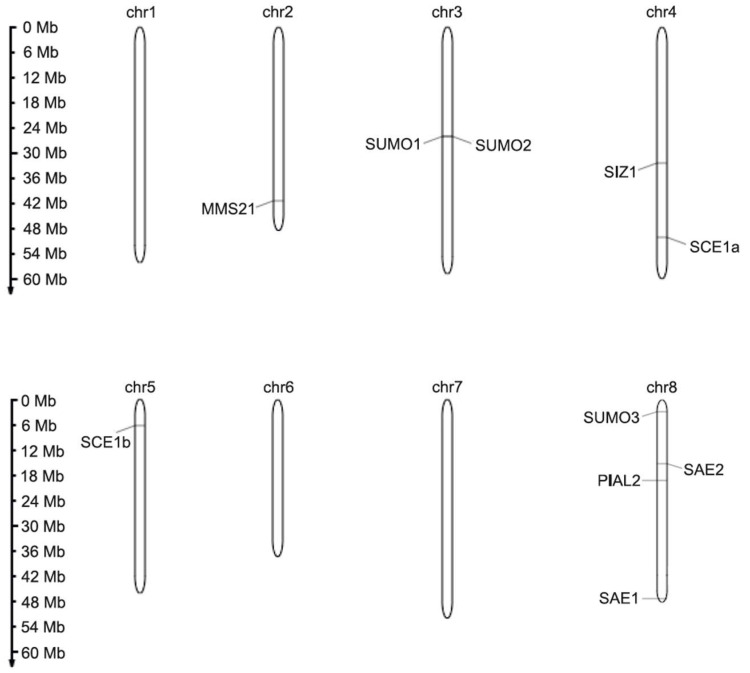
Chromosome distribution of all identified SUMOylation pathway members in *Medicago truncatula*.

In the *M. truncatula* genome, only two genes encoding E1 ligase proteins were identified, namely, Mt SAE1 and Mt SAE2. These proteins clustered with their homologs from *A. thaliana* and *G. max* (Figure 3A). Mt SAE2 clusters more closely with Gm SAE2a than with Gm SAE2b, indicating a stronger phylogenetic relationship between Mt SAE2 and Gm SAE2a. Mt SAE1 is a small subunit consisting of 159 amino acids (Table 1), whereas Mt SAE2 is considerably larger, comprising 635 amino acids. The genes encoding these proteins contain 5 and 11 coding exons, respectively (Figure 2), and both are located on chromosome 8 (Figure 4). Domain analysis revealed that both SAE1 and SAE2 possess a ubiquitin-fold domain (Figure 3B). Additionally, SAE2 contains a cysteine domain. Both domains are essential for the proper function of E1 ligases.

Next in the SUMOylation pathway is the E2 conjugating enzyme. A search of the *M. truncatula* genome identified two genes encoding the SCE1 enzyme, designated Mt *SCE1a* and Mt *SCE1b* (Figure 3A). Mt *SCE1a* and Mt *SCE1b* share high sequence similarity with their homologs in *A. thaliana* and *G. max*, exceeding 87% and 91%, respectively. Both proteins contain a highly conserved Ubiquitin Conjugating Enzyme E2 domain, similar to that of At SCE1 (Figure 3B). In terms of protein structure, Mt SCE1a and Mt SCE1b are both 159 amino acids in length (Table 1) and are encoded by genes comprising five exons each (Figure 2). Despite these similarities, the genes differ in chromosomal localization, with Mt *SCE1a* located on chromosome 4 and Mt *SCE1b* on chromosome 5 (Figure 4).

SUMO modification of target proteins is facilitated by E3 ligase-mediated attachment. In the *M. truncatula* genome, three genes were identified that encode E3 ligases: Mt *MMS21*, Mt *PIAL2*, and Mt *SIZ1*. Phylogenetic analysis showed that these proteins form distinct clades and cluster with their homologs from *A. thaliana* and *G. max* (Figure 3A). All three proteins contain a MIZ-type Zinc Finger domain, which is essential for their interaction with the SUMO–E2 conjugating enzyme (Figure 3B). Notably, SIZ1 proteins from both *A. thaliana* and *M. truncatula* also possess SAP and PDH-type Zinc Finger domains.

The proteins vary in length: Mt PIAL2 consists of 822 amino acids, and Mt SIZ1 of 882 amino acids (Table 1). The genes encoding these proteins comprise 17 coding exons (Figure 2) but are located on different chromosomes—Mt *PIAL2* on chromosome 8 and Mt *SIZ1* on chromosome 4 (Figure 4). In contrast, Mt MMS21 is significantly shorter, with a length of 244 amino acids. The Mt *MMS21* gene contains only seven exons (Figure 2) and is located on chromosome 2 (Figure 4).

### 2.2. Expression Profile SUMO Pathway Genes During Induction Phase of Medicago truncatula Somatic Embryogenesis

To elucidate the potential role and transcriptional regulation of SUMO pathway components in the induction of somatic embryogenesis in *Medicago truncatula*, we analyzed the expression patterns of 10 genes associated with the SUMOylation pathway. Gene expression was assessed in two *M. truncatula* lines—a non-embryogenic line (M9) and an embryogenic line (M9-10a)—at four developmental time points (0, 2, 7, and 14 days) to evaluate their differential responses during somatic embryogenesis induction.

**Figure 5:**
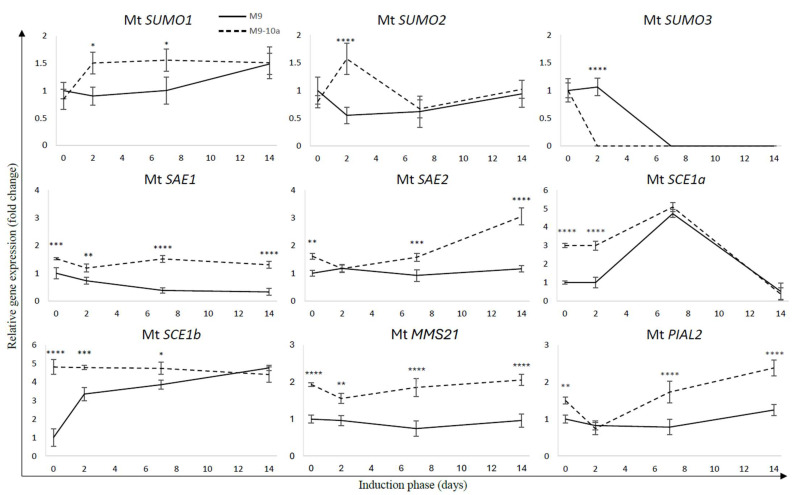
Relative expression patterns of SUMOylation pathway genes in leaf explants of *Medicago truncatula* non-embryogenic (M9) and embryogenic (M9-10a) lines during the induction phase. Gene expression was normalized to *ACTIN2* and presented relative to the lowest observed transcript level, set as 1. Bars represent mean values ± standard deviation (SD) of three independent biological replicates, each with three technical replicates. Solid and dotted lines indicate the M9 and M9-10a lines, respectively. Statistical analysis was performed using two-way ANOVA followed by Tukey’s HSD post hoc test. Asterisks indicate significance levels: * *p*  <  0.05, ** *p*  <  0.01, *** *p*  <  0.001, **** *p*  <  0.0001.

**Figure 6:**
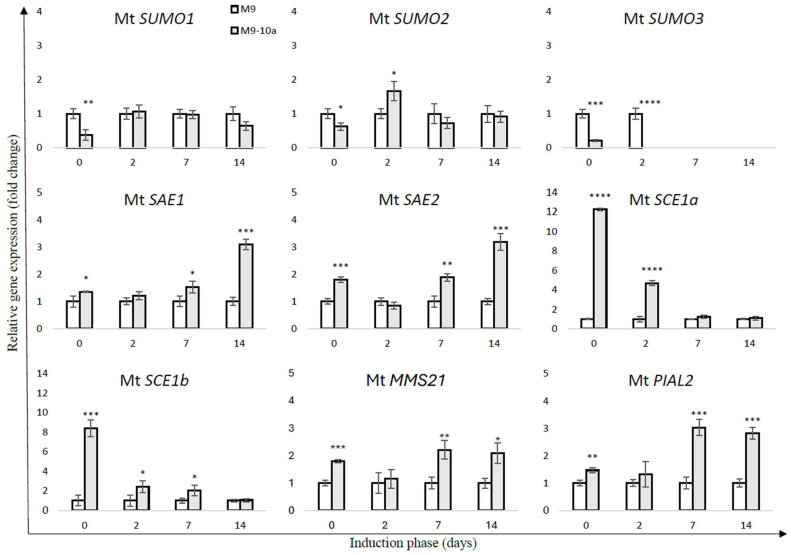
Relative expression of SUMOylation pathway genes during the somatic embryogenesis induction phase in leaf explants of *Medicago truncatula* non-embryogenic (M9) and embryogenic (M9-10a) lines. Gene expression was normalized to *ACTIN2* and presented as fold change relative to the M9 line (set as 1) at each time point. Bars represent mean values ± standard deviation (SD) of three independent biological replicates, each with three technical replicates. Statistical significance between M9 and M9-10a at each time point was determined using Student’s *t*-test. Asterisks indicate significance levels: * *p*  <  0.05, ** *p*  <  0.01, *** *p*  <  0.001, **** *p*  <  0.0001.

Quantitative PCR analysis revealed that most components of the SUMOylation cascade were differentially regulated during the course of somatic embryogenesis (SE). In the M9 line, the expression level of Mt *SUMO1* remained stable until day 7 of SE induction, after which a significant increase of approximately 1.5-fold was observed (Figure 5). Comparative analysis between the M9 and M9-10a lines showed a marked difference in Mt *SUMO1* transcript levels at the initial time point (day 0), with the embryogenic line exhibiting 2.6-fold lower expression compared to the non-embryogenic line (Figure 6). Interestingly, Mt *SUMO1* expression in M9-10a increased sharply—by nearly 1.8-fold—on day 2 and remained at this elevated level through day 14.

A similar expression trend was observed for Mt *SUMO2* (Figure 5). In the M9 line, transcript levels decreased by approximately twofold on day 2 compared to day 0, followed by a slight increase at later stages. In contrast, Mt *SUMO2* expression in the M9-10a line showed a notable twofold increase on day 2, returning to baseline levels by day 14. A direct comparison revealed that at day 0, Mt *SUMO2* expression was 1.25 times higher in the M9 line than in M9-10a. However, on day 2, the embryogenic line displayed a 1.66-fold higher expression than the non-embryogenic line (Figure 6).

In contrast to Mt *SUMO1* and Mt *SUMO2*, Mt *SUMO3* exhibited a distinct expression pattern. In the embryogenic line, Mt *SUMO3* expression was detected only at day 0, with no detectable transcripts at subsequent time points (Figure 5). Conversely, in the M9 line, expression remained stable until day 2, after which it was no longer detectable.

The expression profiles of genes encoding proteins involved in the SUMOylation pathway revealed significant differences between the two analyzed lines. For Mt *SAE1*, the M9 line exhibited a pronounced decrease in expression over time, with transcript levels reduced by approximately threefold by day 14 compared to the initial explant (Figure 5). In contrast, Mt *SAE1* expression in the M9-10a line remained relatively stable throughout the induction phase. A direct comparison between the lines revealed significantly higher transcript levels in the embryogenic line, with the most notable difference observed on day 14, when Mt *SAE1* expression in M9-10a was threefold higher than in the M9 line.

A similar trend was observed for the second SUMO-activating enzyme, Mt SAE2 (Figure 6). In the M9 line, no substantial changes in Mt *SAE2* expression were detected during the induction period (Figure 5). In contrast, in the M9-10a line, Mt *SAE2* expression gradually increased over time, reaching levels nearly 1.9-fold higher on day 14 compared to day 0. On day 14, transcript levels in the embryogenic line were more than threefold higher than in the non-embryogenic line.

In the SUMOylation pathway, the second key component is the SUMO-conjugating enzyme, represented by Mt SCE1a and Mt SCE1b. Analysis of gene expression revealed that transcript levels of Mt *SCE1a* and Mt *SCE1b* were approximately 12-fold and 8-fold higher, respectively, in the embryogenic line compared to the non-embryogenic line at the initial time point (day 0; Figure 6). Notably, Mt *SCE1b* expression in the embryogenic line remained consistently elevated throughout the first week of the induction phase. In contrast, Mt *SCE1a* expression showed a transient increase until day 7, followed by a marked decrease by day 14 (Figure 5). Importantly, Mt *SCE1b* expression remained stable in the M9-10a line across all time points, whereas in the M9 line, its transcript levels increased more than threefold on day 2 compared to day 0.

The final group of enzymes involved in the SUMOylation pathway—the SUMO E3 ligases—includes three proteins: Mt MMS21, Mt PIAL2, and Mt SIZ1. Interestingly, the expression profiles of Mt *MMS21* and Mt *PIAL2* closely resembled those of the SUMO-activating enzymes Mt *SAE1* and Mt *SAE2*, respectively (Figure 5). The genes encoding both ligases exhibited consistently higher expression levels in the embryogenic line compared to the non-embryogenic line throughout the induction phase, with the exception of day 2 (Figure 6). By day 14, expression of Mt *MMS21* and Mt *PIAL2* reached approximately twofold and nearly threefold higher levels, respectively, in the embryogenic line relative to the non-embryogenic line. Notably, no expression of Mt *SIZ1* was detected at any time point during somatic embryogenesis induction in *M. truncatula*.

### 2.3. SUMOylation Site and SIM Prediction

The subsequent phase of this study focused on the identification of SUMOylation sites and SUMO-interacting motifs in proteins encoded by genes that are well-established markers of somatic embryogenesis. These genes include *LEAFY COTYLEDON1* (*LEC1*), *LEAFY COTYLEDON1-like* (*L1L*), *LEC2*, *BABY BOOM* (*BBM*), *WUSCHEL* (*WUS*), *WUSCHEL-Related Homeobox 5* (*WOX5*), *SHOOT MERISTEMLESS* (*STM*), *SOMATIC EMBRYO-RELATED FACTOR1* (*SERF1*), *SOMATIC EMBRYOGENESIS RECEPTOR KINASE1* (*SERK1*), *ABA INSENSITIVE3* (*ABI3*), *FUSCA3* (*FUS3*), and *AGAMOUS-like15* (*AGL15*). The aim of this analysis was to elucidate the potential role of SUMOylation in regulating the activity of these proteins, which are recognized as key regulators of somatic embryogenesis.

The analyses revealed that out of the 12 selected proteins, 10 were predicted to contain SUMOylation sites (Table S2). Notably, eight of these proteins were predicted to harbor two or more such sites, with *LEC1*, *BBM*, *STM*, *SERF1*, *ABI3*, and *FUS3* showing particularly high prediction scores—exceeding 0.9—in at least two independent SUMOylation prediction tools. These findings suggest that these proteins are likely subject to post-translational regulation via SUMO modification.

Among the proteins predicted to possess SUMOylation sites, seven also contained SUMO-interacting motifs (SIMs), indicating their potential involvement in SUMO-mediated signaling pathways.

In contrast, although no SUMOylation sites were predicted for *LEC2* and *L1L*, our analysis identified putative SIMs within their sequences, suggesting that they may still participate in SUMO-dependent interactions.

## 3. Discussion

The remarkable regenerative capacity of plants, which enables them to survive adverse environmental conditions and regenerate from tissue fragments, has captivated scientists for decades. A prime example of this regenerative ability is somatic embryogenesis (SE), a process characterized by the formation of embryos from somatic cells of explants. The transition from a vegetative developmental program to an embryonic one entails extensive physiological, biochemical, and molecular changes. Years of research have demonstrated that these changes involve alterations in the expression of numerous gene groups, many of which are essential for successful SE progression.

In recent years, there has been growing interest in the epigenetic modifications that accompany this process. It is now well established that proper epigenetic regulation is critical for the induction of the embryonic program. One genetic mechanism that remains underexplored is SUMOylation, a post-translational modification involving small ubiquitin-like modifier (SUMO) proteins. Previous studies have shown that SUMOylation regulates various aspects of plant development and plays a key role in plant responses to environmental stress. Therefore, the aim of this study was to investigate the potential role of SUMO proteins in somatic embryogenesis in *Medicago truncatula*, thereby advancing our understanding of the regulatory mechanisms underpinning this complex developmental process.

Through comparative analysis of amino acid sequences of proteins involved in the SUMOylation pathway in *Arabidopsis thaliana* and *Glycine max*, we identified, for the first time, a total of 10 homologs genes in *Medicago truncatula*. Among these, three genes encode SUMO proteins, while the remaining seven are associated with various enzymatic functions. Phylogenetic analysis by Li et al. [24] classified SUMO proteins into two distinct groups: canonical and non-canonical. Our findings support this classification, confirming that the canonical group includes SUMO1-2 proteins from *A. thaliana* and *M. truncatula*, as well as SUMO1-3 from *G. max*. The non-canonical group comprises Mt SUMO3, which exhibits greater similarity to *G. max* proteins than to those from Arabidopsis. Enzymes involved in the SUMOylation cascade cluster closely with their homologs in *G. max* and *A. thaliana*. Additionally, analysis of the distribution and presence of key functional domains essential for protein activity revealed a high degree of conservation between *A. thaliana* and *M. truncatula*.

In addition, gene structure analysis revealed distinct architectures between the two groups of Mt SUMO proteins: canonical SUMOs are encoded by genes containing three exons, whereas the non-canonical group exhibits a four-exon structure. Conserved motif analysis using MEME demonstrated similar motif compositions across all SUMO proteins from *M. truncatula* and *A. thaliana*, supporting the observed phylogenetic relationships. These structural and motif differences likely reflect functional divergence and evolutionary adaptation within the SUMO protein family, suggesting distinct roles during plant development and stress responses.

In *A. thaliana*, SUMO1 and SUMO2 can form poly-SUMO chains due to the presence of internal SUMOylation motifs, whereas SUMO3 lacks this motif, preventing such chain formation [25,26]. These *SUMO* proteins exhibit distinct expression profiles across various tissues. Specifically, *SUMO1* and *SUMO2* show partially overlapping expression patterns in leaves, while *SUMO3* differs significantly from both, particularly in developing flowers [22,23]. During embryonic development, At *SUMO1* is expressed from the early stages, including the globular, heart, and torpedo stages, whereas At *SUMO2* expression begins later, at the late heart stage. Although both At *SUMO1* and At *SUMO2* are constitutively expressed, their spatial expression patterns do not overlap. In contrast, At *SUMO3* is not expressed in seeds or embryos; its expression is restricted to hydathodes and the vasculature of mature leaves. Notably, At *SUMO3* transcription was detected only in the primary explant on the second day of somatic embryogenesis induction. In later stages, its expression was likely suppressed due to advanced cell dedifferentiation and the progressive loss of vegetative identity. This observation aligns with the findings of van den Burg et al. [23], who reported that SUMO3 is present in mature leaves but absent in embryonic tissues.

Moreover, differential transcript levels of small SUMO subunits have been observed during the induction of somatic embryogenesis in *M. truncatula*. Our results indicate that the genes encoding SUMO proteins (Mt SUMO1-3) exhibit distinct expression patterns throughout this process. Notably, the increased expression of Mt *SUMO1* and Mt *SUMO2* during the early stages of somatic embryogenesis in the M9-10a line suggests their potential involvement in initiating embryonic development, likely through the modification of key regulatory proteins that facilitate cellular reprogramming. Conversely, the reduced expression levels of Mt *SUMO1* and Mt *SUMO2* in the M9 line may reflect an impaired ability to transition into an embryogenic state, highlighting the critical roles these proteins play in the embryogenic program. Supporting this notion, similar observations have been reported in Arabidopsis *sum1sum2* double mutants. These mutants, which lack accumulation of the essential SUMO proteins SUMO1 and SUMO2, exhibit lethality due to the absence of viable zygotic embryos. In contrast, single mutants for either SUMO1 or SUMO2 do not show any phenotypic abnormalities [22].

Studies have shown that not only the small SUMO subunits but also the enzymatic components of the SUMOylation pathway—including E1 ligases (Mt SAE1 and Mt SAE2), E2 conjugating enzymes (Mt SCE1a and Mt SCE1b), and E3 ligases (Mt MMS21, Mt PIAL2, and Mt SIZ1)—display distinct expression patterns between the non-embryogenic (M9) and embryogenic (M9-10a) lines during somatic embryogenesis. The M9 line generally exhibits lower and often declining expression levels, suggesting insufficient signaling required for successful embryogenesis. In contrast, the M9-10a line shows higher initial expression levels with notable increases at critical time points, particularly on days 7 and 14, highlighting the importance of these components in initiating the embryogenic program. Notably, expression of key somatic embryogenesis genes such as *ABI3*, *FUS3*, *LEC1*, *L1L*, *LEC2*, and *STM* was also detected at these time points [7,27].

Moreover, studies by Saracco et al. [22] demonstrated that *A. thaliana* mutants lacking SAE2 (*sae2*) and SCE1 (*sce1*) exhibited embryo lethality, with developmental arrest occurring at early embryonic stages, including the globular, heart, and early torpedo phases. Gene expression analysis during somatic embryogenesis induction in *M. truncatula* revealed that Mt *SAE2* transcript levels increased progressively over the induction period in the embryogenic line, suggesting its involvement in initiating the embryogenic program within callus cells. In contrast, the absence of such transcriptional changes in the non-embryogenic line correlates with its inability to undergo embryogenesis.

Similarly, Mt *SAE1*, another key component of the E1-activating enzyme complex required to initiate SUMO conjugation, displayed stable expression in the embryogenic line but showed a marked decline in the non-embryogenic line. This downregulation in M9 suggests a disruption in the SUMOylation pathway, as the activation step mediated by SAE1 is essential for transferring SUMO proteins to downstream E2 and E3 enzymes. Without this step, SUMO cannot be covalently attached to target proteins, thereby impairing the post-translational regulation of key factors involved in embryogenic reprogramming. The maintained expression of *Mt SAE1* in the embryogenic line likely ensures functional SUMOylation, supporting cellular plasticity and the acquisition of embryogenic competence.

In contrast, Mt *SCE1a* and Mt *SCE1b* exhibited higher expression levels during the early stages of the induction phase in the embryogenic line compared to the non-embryogenic line, up to day 7. However, as induction progressed, the transcript levels of these genes converged, with increased expression observed in the non-embryogenic line as well. This pattern may indicate a potential role for Mt *SCE1a* and Mt *SCE1b* in the dedifferentiation of explant cells. Overall, these findings suggest that both E1 and E2 components are crucial for the induction of somatic embryogenesis and, consequently, for successful embryo formation.

Interestingly, differences in the transcript levels of genes encoding SUMO pathway components were already apparent in the initial explants, indicating that regulation of the SUMO pathway begins early in the embryogenic process. Similar patterns have been observed for other epigenetic regulators, including Polycomb and Trithorax group proteins [7,8]. Moreover, in leaves harvested directly from parent plants, significant expression differences were noted between the non-embryogenic (M9) and embryogenic (M9-10a) lines. Specifically, small SUMO subunits exhibited higher expression in the non-embryogenic line, whereas other proteins showed the opposite trend, with elevated expression in the embryogenic line.

The role of SUMOylation in mediating environmental stress responses is well established; however, its specific regulatory function during the early stages of somatic embryogenesis remains an area of considerable interest. Our data suggest that SUMOylation is closely linked to the developmental transitions occurring throughout SE. For example, the temporal regulation and expression stability of SUMO-activating and conjugating enzymes (Mt SAE1, SAE2, SCE1a, and SCE1b) imply essential roles in modulating the SUMOylation status of target proteins during the SE process. Notably, elevated expression levels of these enzymes in the embryogenic M9-10a line correlate with its enhanced embryogenic capacity.

Furthermore, we extended our analysis to predict SUMOylation sites and SUMO-interacting motifs (SIMs) in key SE marker proteins. Our findings indicate that all examined proteins harbor SUMOylation and/or SIM sites, suggesting that SUMOylation may modulate their interactions with other signaling molecules or transcriptional networks during SE induction in *M. truncatula*. These results support the hypothesis that SUMOylation functions as a molecular switch regulating the activity of critical regulators, thereby influencing the somatic embryogenesis program.

In conclusion, our results establish a strong link between the SUMOylation pathway and the regulation of somatic embryogenesis in *Medicago truncatula*. The differential expression of SUMO-related genes in embryogenic versus non-embryogenic lines, combined with the predicted presence of SUMOylation sites in key developmental regulators, underscores the potential role of SUMOylation as a critical regulatory mechanism during SE. Despite this emerging connection, the precise molecular mechanisms through which SUMOylation modulates gene expression and initiates embryogenesis remain to be fully elucidated. Future studies focused on identifying the specific SUMOylation targets and their downstream effects on signaling networks will be essential for advancing our understanding of how SUMOylation orchestrates plant developmental processes, particularly somatic embryogenesis.

## 4. Materials and Methods

### 4.1. Tissue Culture Protocol

Tissue culture experiments were performed using the non-embryogenic (M9) and embryogenic (M9-10a) lines of *Medicago truncatula* cv. Gaertn., following the protocol previously described by Orłowska et al. [7]. For the production of donor plants, seeds from these lines were utilized, which were kindly provided by Professor Pedro Fevereiro from ITQB Universidade Nova de Lisboa, Lisbon, Portugal [28].

### 4.2. Identification of SUMO Pathway Genes in Medicago truncatula Genome

The amino acid sequences of *Arabidopsis thaliana* SUMOylation pathway proteins were obtained from the TAIR database (https://www.arabidopsis.org/ (accessed on 1 July 2024)) and used as queries for BLASTp searches against the *Medicago truncatula* genome in the NCBI database (accessed on 1 July 2024). Homologous genes were selected based on stringent criteria: E-value ≤ 1 × 10^−20^, sequence identity ≥ 40%, and query coverage ≥ 70%. The full-length protein sequences of identified candidates were aligned using the ClustalW algorithm in Geneious 6.1 (https://www.geneious.com (accessed on 1 July 2024)) to assess sequence conservation. Phylogenetic relationships were inferred using the Neighbor-Joining method with 1000 bootstrap replicates. Conserved domains characteristic of E1, E2, and E3 SUMOylation enzymes were verified using the InterPro database (https://www.ebi.ac.uk/interpro/ (accessed on 3 July 2024)).

### 4.3. Gene Structure, Motif Identification, and Chromosomal Distribution

The exon and intron structures of the identified genes were analyzed by comparing the coding sequences (CDS) with their corresponding genomic sequences retrieved from the NCBI database. Visualization was performed using the Gene Structure Display Server (https://gsds.gao-lab.org/ (accessed on 15 July 2024)) [29]. Motif analysis of SUMO proteins was conducted using MEME version 5.5.4 (https://meme-suite.org/ (accessed on 16 July 2024)) [30] with default parameters, except for the following settings: minimum motif width set to 8 amino acids and the maximum number of motifs to be identified limited to 55. The distribution of identified SUMO proteins was determined based on genome annotation data and visualized using the MapGene2Chromosome v2.1 tool (http://mg2c.iask.in/mg2c_v2.1/ (accessed on 16 July 2024)) [31].

### 4.4. Gene Expression Analysis

Total RNA was extracted from plant material collected at 0, 2, 7, and 14 days of the somatic embryogenesis induction phase. Explants were incubated on induction medium supplemented with 1 µM zeatin and 0.5 µM 2,4-D, as previously described [8]. Primer sequences for genes encoding SUMO pathway members are listed in Table S1. Gene expression levels were normalized to *ACTIN2*, and relative expression was calculated using the 2^−∆∆Ct^ method. Each sample consisted of three biological replicates, each with three technical replicates. Data are presented as mean values ± standard deviation (SD). Statistical analysis was performed using Student’s *t*-test for pairwise comparisons between M9 and M9-10a at each time point, and two-way ANOVA followed by Tukey’s HSD post hoc test for time course comparisons. Differences were considered statistically significant at *p* < 0.05 or *p* < 0.01.

### 4.5. Prediction of SUMOylation and SIM Sites in Key Proteins Involved in Medicago truncatula Somatic Embryogenesis

Analysis of SUMOylation and SUMO-interacting motifs (SIMs) in key proteins involved in somatic embryogenesis of *Medicago truncatula*, including *LEC1*, *L1L*, *LEC2*, *BBM*, *WUS*, *WOX5*, *STM*, *SERF1*, *SERK1*, *ABI3*, *FUS3*, and *AGL15*, was performed using bioinformatics tools. Protein sequences for these regulators were retrieved from the NCBI database. Potential SUMOylation sites were predicted using GPS-SUMO 2.0 (https://sumo.biocuckoo.cn/ (accessed on 20 August 2024)) [32], which identifies possible SUMO attachment motifs based on established consensus sequences. SIMs were analyzed using the JASSA platform (http://www.jassa.fr/ (accessed on 20 August 2024)) [33], which predicts motifs based on sequence patterns and structural features. Additionally, the SUMOplot™ Analysis Program (https://www.abcepta.com/sumoplot (accessed on 20 August 2024)) was employed to validate and visualize the predicted SUMOylation sites and SIMs, providing confidence scores for each motif. All analyses were conducted following the respective software protocols (version as available at the time of access). Predicted sites were considered significant based on their confidence scores and the consensus among the tools.

## Figures and Tables

**Table 1:** Characterization of *Medicago truncatula* SUMOylation pathway proteins. Locus ID for *Mt SUMO3* is not available in the NCBI database; genomic coordinates are provided instead. Protein length is given in amino acids (aa).

Group	Gene Name	Locus ID	Chromosome	Gene Accession Number	Exon No.	Protein Accession Number	Protein Length (aa)
SUMO	Mt *SUMO1*	MtrunA17_Chr3g0099761	3	XM_013604411.3	3	XP_013459865.1	101
	Mt *SUMO2*	MtrunA17_Chr3g0099771	3	XM_013604412.3	3	XP_013459866.1	101
	Mt *SUMO3*	-	8	XM_039829461.1	4	XP_039685395.1	110
E1	Mt *SAE1*	MtrunA17_Chr8g0386251	8	XM_013591535.3	11	XP_013446989.2	325
	Mt *SAE2*	MtrunA17_Chr8g0352371	8	XM_013589621.3	11	XP_013445075.1	635
E2	Mt *SCE1a*	MtrunA17_Chr4g0053191	4	XM_013601981.3	5	XP_013457435.1	159
	Mt *SCE1b*	MtrunA17_Chr5g0401831	5	XM_003611630.4	5	XP_003611678.1	159
E3	Mt *MMS21*	MtrunA17_Chr2g0316861	2	XM_013609188.3	7	XP_013464642.1	244
	Mt *PIAL2*	MtrunA17_Chr8g0356491	8	XM_039828859.1	17	XP_039684793.1	822
	Mt *SIZ1*	MtrunA17_Chr4g0029701	4	XM_039833913.1	17	XP_003606454.1	882

## Data Availability

The additional data supporting the manuscript are available from the corresponding author upon request.

## Supplementary Materials

The following supporting information can be downloaded at https://www.mdpi.com/article/10.3390/ijms26178133/s1.

## Abbreviations

The following abbreviations are used in this manuscript:

DSE	Direct somatic embryogenesis
ISE	Indirect somatic embryogenesis
M9	Non-embryogenic line
M9-10a	Embryogenic line
PEMs	Pro-embryogenic masses
PGRs	Plant growth regulators
SE	Somatic embryogenesis
SIM	SUMO-interacting motif
SUMO	Small ubiquitin-like modifier
