# Supplementary material for: Identification of SUMO Proteins and Their Expression Profile During Induction of Somatic Embryogenesis in Medicago truncatula Gaertn
Source: Int J Mol Sci. 2025 Aug 22;26(17):8133. doi: 10.3390/ijms26178133 (PMC12428226; doi:10.3390/ijms26178133)
Supplement: Supplementary file 1 [file ijms-26-08133-s001.zip › Supplementary table S1.pdf]

Table S1. Primers sequences used for qPCR analysis of *M. truncatula* SUMOylation pathway genes

Gene name	Primer sequence
SUMO1	**F:** ATCGTCGCCGTGATTCTACA<br>**R:** CCACCTGCTTCTGGCTTCTT
SUMO2	**F:** CATAGCACAATTGTCGGCGG<br>**R:** TGTCTTCTTCGGTGTTGGGG
SUMO3	**F:** TGCCTTCATTCTCCCACATCC<br>**R:** GTCTGTGCATACAAAGGACAACA
SUMO4	**F:** CCCACATCCCTTGATATGAGCA<br>**R:** CCCATCCTGAGCCCTAATTGA
SAE1	**F:** TCAACTTCCACTCACCGCAA<br>**R:** TTCCTGCGCCGTTAACTCTT
SAE2	**F:** ATGGACTCAACCAGTCGCAG<br>**R:** ATTGGTGCGTCTGAGGTGTT
SCE1a	**F:** TCTGGTATTGCTCGTGGTCG<br>**R:** AACCATCAGCTGCTGTCTCC
SCE1b	**F:** AGTCAAGCAGCAAGCAAAGC<br>**R:** AGCACCGAGCAAGAAATGGT
MMS21	**F:** CCAAAATTCCCCTTTCGCGG<br>**R:** TTATCCGACCAGAAACGCCG
PIAL2	**F:** GTATCGCCGTCGCTAGTCAA<br>**R:** GTCAGTTGGATTGCCGGGTA
SIZ1	**F:** CTGCCGCGTGATTTCAACAA<br>**R:** TTCCACAAAACCGTAATAAGAATGA
