# Supplementary material for: Identification of SUMO Proteins and Their Expression Profile During Induction of Somatic Embryogenesis in Medicago truncatula Gaertn
Source: Int J Mol Sci. 2025 Aug 22;26(17):8133. doi: 10.3390/ijms26178133 (PMC12428226; doi:10.3390/ijms26178133)
Supplement: Supplementary file 1 [file ijms-26-08133-s001.zip › Supplementary table S2.pdf]

Table S2. Predicted SUMOylation and SIM sites in key proteins involved in *Medicago truncatula* somatic em[illegible]

Protein name	Accession number	SUMOylation site					[illegible]	
		Position (aa)	Peptide	Prediction score			Position site (aa)	Sequenc[illegible]
				Jassa	GPS-SUMO	SUMOplot		
LEC1	XP_003589766.1	182	NFLHL**<u>K</u>RD**NHNN	low	0.9147	0.91	16-19	PIAN**VIRI**[illegible]
L1L	XP_013458658.1						68-71	PIAN**VIRI**[illegible]
LEC2	XP_013457003.3						92-95	TPMS**PILV**[illegible] D
BBM	XP_003624212.1	113	QQAQ**PKLE**NFLG	high	0.9277	0.61	206-212	GSQS**SSSV**[illegible] ANV
		306	GYDK**EEKA**ARAY	low	0.8505	0.5		
WUS	XP_003612158.1	119	SAAN**WKPD**HHEQ	none	0.9001	0.64	97-101	FTSD**VNVV**[illegible] R
							99-102	SDVN**VVPI**[illegible]
							100-103	DVNV**VPII**[illegible] P
WOX5	XP_003616581.1	42	TTEQ**VKLL**TELF	none	0.8597			
		72	LSFY**GKIE**SKNV	none		0.67		
STM	XP_013462900.1	118	SSSS**VKSK**IMAH	none	0.8311			
		189	CEML**IKYE**QELS	high		0.94		
		258	EDRE**LKGQ**LLRK	none	0.8896			
		273	YLGS**LKQE**FMKK	low	0.8705			
SERF1	XP_003589850.2	31	FLHP**VKLENPLPEF** **D**F	high	0.9086	0.93		
		83	PSKE**IKDE**VAIA	high		0.94		
		100	SKKV**KKEE**ECVD	Low		0.93		
		177	AGVC**DPKP**NSCG	low	0.9623			
SERK1	AAN64293.1	335	AVKR**LKEE**RTPG	Low	0.8445	0.91	73-76	NDNS**VIRV**[illegible] N

		426	HDHC**DPKI**IHRD	Low	0.8588		171-174	SALQ**VLDL**SNNQ	SIM Type 1	4.404	
		433	IIHR**DVKA**ANIL	Low	0.9437		556-559	QLIQ**VALL**CTQG	SIM Type 2	0.413	
		532	LLKE**KKLE**MLVD	low		0.48					
ABI3	XP_039683994.1	109	SWAV**LKSDVEVDHQG**Y	Low	0.9358	0.91	160-164	SNND**VVVGG**GDCM			0.9202
		504	GPSA**TKEA**RKK	none	0.8248		708-711	QEGD**FIVI**YSDV	SIM Type 4	0.961	0.8905
		615	RRQG**WKPE**KNLR	none	0.8575	0.64					
		643	RIVL**PKKEAET**HLPE	high		0.61					
FUS3	XP_003624470.1	16	KVVV**QKTE**ACGF		0.9357	0.5	12-16	GKKK**VVVQK**TEAC			0.9382
		36	GFVN**VKGD**NNNG	high	0.8973	0.93					
AGL15	XP_013443646.1	84	ASVE**YKTE**ILPK	none	0.9056		43-46	CDAE**VAVI**IFSN	SIM Type α	0.998	0.8985
		90	TEIL**PKED**SRMV	Low		0.61	96-99	DSRM**VEIL**KDEI	SIM Type 2	0.918	
		100	MVEI**LKDE**IAKL	Low	0.8430	0.91					
		105	KDEI**AKLE**TNQL	Low		0.79					
		221	RNCS**DEKA**DSDT	low	0.8955	0.5					
